# 1,3-Bis(2-anilino-2-oxoeth­yl)-1*H*-imidazol-3-ium chloride

**DOI:** 10.1107/S1600536812027110

**Published:** 2012-06-27

**Authors:** Chuang-Yi Liao, Hon Man Lee

**Affiliations:** a National Changhua University of Education, Department of Chemistry, Changhua, Taiwan 50058

## Abstract

In the cation of the title salt, C_19_H_19_N_4_O_2_^+^·Cl^−^, the dihedral angles between the imidazole ring and the phenyl rings are 70.39 (8) and 86.26 (9)°. The chloride anion inter­acts with the cation through an N—H⋯Cl hydrogen bond. In the crystal, classical N—H⋯O hydrogen bonds link the cations into chains parallel to the *b* axis. Non-classical C—H⋯Cl and C—H⋯O hydrogen bonds further connect the chains into a three-dimensional network.

## Related literature

For the crystal structure of an acetonitrile monosolvate deriv­ative of the title compound, see: Liao & Lee (2011 ▶). For the crystal structures of nickel, palladium, and silver complexes with ligands derived from the title compound, see: Liao, Chan, Chang *et al.* (2007 ▶); Liao, Chan, Zeng *et al.* (2007 ▶); Liao *et al.* (2008 ▶).
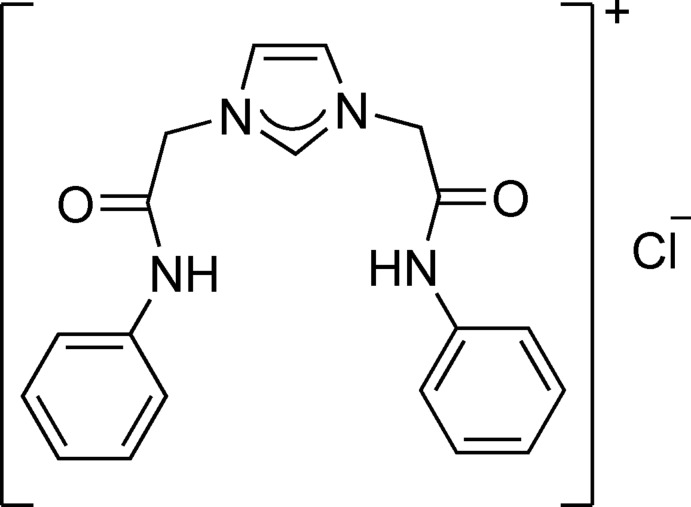


## Experimental

### 

#### Crystal data

C_19_H_19_N_4_O_2_^+^·Cl^−^
*M* *_r_* = 370.83
Monoclinic, 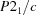

*a* = 8.4375 (5) Å
*b* = 12.0446 (7) Å
*c* = 17.5449 (10) Å
β = 90.789 (3)°
*V* = 1782.85 (18) Å^3^
*Z* = 4
Mo *K*α radiation
μ = 0.24 mm^−1^
*T* = 150 K
0.11 × 0.09 × 0.07 mm

#### Data collection

Bruker SMART APEXII diffractometer
Absorption correction: multi-scan (*SADABS*; Sheldrick, 2003 ▶) *T*_min_ = 0.975, *T*_max_ = 0.984
11822 measured reflections
3684 independent reflections
1924 reflections with *I* > 2σ
*R*_int_ = 0.062

#### Refinement

*R*[*F* ^2^ > 2σ(*F* ^2^)] = 0.046
*wR*(*F* ^2^) = 0.096
*S* = 0.92
3684 reflections
311 parameters
All H-atom parameters refined
Δρ_max_ = 0.25 e Å^−3^
Δρ_min_ = −0.22 e Å^−3^

### 

Data collection: *APEX2* (Bruker, 2007 ▶); cell refinement: *SAINT* (Bruker, 2007 ▶); data reduction: *SAINT*; program(s) used to solve structure: *SHELXTL* (Sheldrick, 2008 ▶); program(s) used to refine structure: *SHELXTL*; molecular graphics: *SHELXTL*; software used to prepare material for publication: *DIAMOND* (Brandenburg, 2006 ▶).

## Supplementary Material

Crystal structure: contains datablock(s) I, global. DOI: 10.1107/S1600536812027110/rz2771sup1.cif

Structure factors: contains datablock(s) I. DOI: 10.1107/S1600536812027110/rz2771Isup2.hkl

Supplementary material file. DOI: 10.1107/S1600536812027110/rz2771Isup3.cml

Additional supplementary materials:  crystallographic information; 3D view; checkCIF report

## Figures and Tables

**Table 1:** Hydrogen-bond geometry (Å, °)

*D*—H⋯*A*	*D*—H	H⋯*A*	*D*⋯*A*	*D*—H⋯*A*
N3—H3*A*⋯O2^i^	0.87 (2)	1.94 (2)	2.799 (3)	171 (2)
N4—H4*A*⋯Cl1^ii^	0.99 (2)	2.19 (2)	3.182 (2)	177.4 (19)
C1—H1⋯Cl1^iii^	0.97 (2)	2.58 (2)	3.482 (3)	155.0 (16)
C2—H2⋯O1^ii^	0.91 (2)	2.32 (2)	3.082 (3)	140.6 (17)
C13—H13⋯O2	0.97 (2)	2.29 (2)	2.862 (3)	116.9 (16)
C4—H24*B*⋯O2^i^	0.98 (2)	2.54 (2)	3.308 (3)	136.0 (16)

Symmetry codes: (i) 
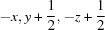
; (ii) 
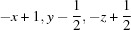
; (iii) 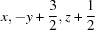.

**Fig. 1.**
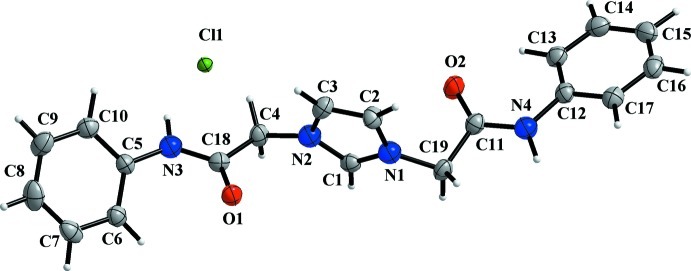
The structure of the title compound, showing 50% probability displacement ellipsoids for the non-hydrogen atoms. The H atoms are dipicted by circles of an arbitrary radius.

**Fig. 2.**
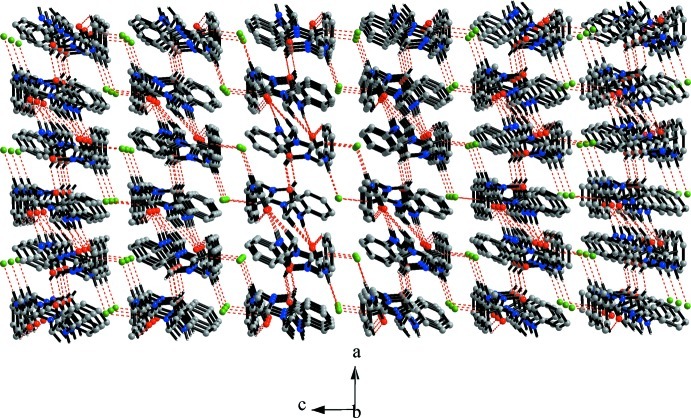
A view of the crystal packing along the b axis, displaying the hydrogen bonds as dashed lines.

## supplementary crystallographic information

### Comment

The crystal structure of a related acetonitrile monosolvate was reported by us previously (Liao & Lee 2011). The compound is a good precursor for the preparation of transition metal complexes of N-heterocyclic carbene (NHC) ligands. Nickel (Liao, Chan, Chang *et al.* 2007), palladium (Liao, Chan, Zeng *et al.* 2007) and silver (Liao *et al.* 2008) complexes with NHC ligands derived from the title compound were successfully prepared.

The structure of the title compound is shown in Fig. 1. The chloride anion forms a hydrogen bond with one of the amido H-atoms (Table 1). In the cation, the dihedral angles formed by the imidazole ring with the C5–C10 and C12–C17 phenyl rings are 70.39 (8) and 86.26 (9)°, respectively. Classical N—H···O hydrogen bonds link the cations into chains parallel to the *b* axis. Non-classical hydrogen bonds of the type C—H···Cl and C—H···O further connects the chains into a three-dimensional network (Fig. 2).

### Experimental

The compound was prepared according to the literature method (Liao, Chan, Zeng *et al.* 2007). Crystals suitable for X-ray analysis were obtained by slow diffusion of diethyl ether into a dichloromethane solution of the compound at room temperature.

### Refinement

All the hydrogen atoms were located in a difference Fourier map and freely refined.

### Crystal data

**Table:**

C_19_H_19_N_4_O_2_^+^·Cl^−^	*F*(000) = 776
*M**_r_* = 370.83	*D*_x_ = 1.382 Mg m^−^^3^
Monoclinic, *P*2_1_/*c*	Mo *K*α radiation, λ = 0.71073 Å
Hall symbol: -P 2ybc	Cell parameters from 1122 reflections
*a* = 8.4375 (5) Å	θ = 2.9–18.5°
*b* = 12.0446 (7) Å	µ = 0.24 mm^−^^1^
*c* = 17.5449 (10) Å	*T* = 150 K
β = 90.789 (3)°	Prism, colourless
*V* = 1782.85 (18) Å^3^	0.11 × 0.09 × 0.07 mm
*Z* = 4	

### Data collection

**Table:**

Bruker SMART APEXII diffractometer	3684 independent reflections
Radiation source: fine-focus sealed tube	1924 reflections with *I* > 2σ
Graphite monochromator	*R*_int_ = 0.062
ω scans	θ_max_ = 26.5°, θ_min_ = 2.1°
Absorption correction: multi-scan (*SADABS*; Sheldrick, 2003)	*h* = −10→10
*T*_min_ = 0.975, *T*_max_ = 0.984	*k* = −11→15
11822 measured reflections	*l* = −21→21

### Refinement

**Table:**

Refinement on *F*^2^	Primary atom site location: structure-invariant direct methods
Least-squares matrix: full	Secondary atom site location: difference Fourier map
*R*[*F*^2^ > 2σ(*F*^2^)] = 0.046	Hydrogen site location: inferred from neighbouring sites
*wR*(*F*^2^) = 0.096	All H-atom parameters refined
*S* = 0.92	*w* = 1/[σ^2^(*F*_o_^2^) + (0.0371*P*)^2^] where *P* = (*F*_o_^2^ + 2*F*_c_^2^)/3
3684 reflections	(Δ/σ)_max_ = 0.004
311 parameters	Δρ_max_ = 0.25 e Å^−^^3^
0 restraints	Δρ_min_ = −0.22 e Å^−^^3^

### Special details

**Table:**

Geometry. All e.s.d.'s (except the e.s.d. in the dihedral angle between two l.s. planes) are estimated using the full covariance matrix. The cell e.s.d.'s are taken into account individually in the estimation of e.s.d.'s in distances, angles and torsion angles; correlations between e.s.d.'s in cell parameters are only used when they are defined by crystal symmetry. An approximate (isotropic) treatment of cell e.s.d.'s is used for estimating e.s.d.'s involving l.s. planes.
Refinement. Refinement of *F*^2^ against ALL reflections. The weighted *R*-factor *wR* and goodness of fit *S* are based on *F*^2^, conventional *R*-factors *R* are based on *F*, with *F* set to zero for negative *F*^2^. The threshold expression of *F*^2^ > σ(*F*^2^) is used only for calculating *R*-factors(gt) *etc*. and is not relevant to the choice of reflections for refinement. *R*-factors based on *F*^2^ are statistically about twice as large as those based on *F*, and *R*- factors based on ALL data will be even larger.

### Fractional atomic coordinates and isotropic or equivalent isotropic displacement parameters (Å^2^)

**Table:**

	*x*	*y*	*z*	*U*_iso_*/*U*_eq_	
C1	0.2603 (3)	0.6481 (2)	0.34316 (15)	0.0272 (6)	
C2	0.3583 (3)	0.5510 (2)	0.24844 (14)	0.0311 (6)	
C3	0.2750 (3)	0.6355 (2)	0.21849 (16)	0.0320 (7)	
C4	0.1131 (3)	0.7918 (2)	0.26932 (16)	0.0302 (6)	
C5	0.1673 (3)	1.0668 (2)	0.17161 (13)	0.0244 (6)	
C6	0.2754 (3)	1.1355 (2)	0.20752 (15)	0.0306 (6)	
C7	0.3198 (3)	1.2344 (2)	0.17338 (17)	0.0368 (7)	
C8	0.2524 (3)	1.2655 (3)	0.10441 (17)	0.0449 (8)	
C9	0.1429 (3)	1.1971 (2)	0.06952 (16)	0.0421 (8)	
C10	0.1013 (3)	1.0980 (2)	0.10172 (14)	0.0331 (7)	
C11	0.3070 (3)	0.3735 (2)	0.37508 (13)	0.0295 (6)	
C12	0.3050 (3)	0.1740 (2)	0.40765 (12)	0.0283 (6)	
C13	0.1423 (3)	0.1560 (2)	0.40360 (13)	0.0322 (7)	
C14	0.0841 (4)	0.0495 (2)	0.41476 (14)	0.0392 (7)	
C15	0.1843 (4)	−0.0380 (2)	0.42981 (14)	0.0405 (7)	
C16	0.3452 (4)	−0.0205 (2)	0.43115 (14)	0.0386 (7)	
C17	0.4066 (3)	0.0855 (2)	0.42045 (13)	0.0328 (7)	
C18	0.2067 (3)	0.8904 (2)	0.23976 (13)	0.0274 (6)	
C19	0.4067 (3)	0.4786 (2)	0.38240 (16)	0.0314 (7)	
Cl1	0.26726 (7)	0.80315 (6)	0.03880 (3)	0.0388 (2)	
H1	0.231 (2)	0.6733 (16)	0.3934 (11)	0.022 (6)*	
H2	0.416 (3)	0.4943 (18)	0.2284 (12)	0.028 (7)*	
H3	0.264 (3)	0.6624 (18)	0.1704 (13)	0.034 (7)*	
H6	0.320 (3)	1.1175 (18)	0.2553 (12)	0.033 (7)*	
H7	0.392 (3)	1.2781 (18)	0.2008 (12)	0.032 (7)*	
H8	0.282 (3)	1.3315 (19)	0.0838 (13)	0.035 (7)*	
H9	0.091 (3)	1.2152 (19)	0.0257 (13)	0.042 (8)*	
H10	0.023 (2)	1.0480 (17)	0.0767 (11)	0.027 (6)*	
H13	0.068 (3)	0.2155 (18)	0.3914 (11)	0.028 (6)*	
H14	−0.026 (3)	0.0440 (19)	0.4129 (13)	0.043 (8)*	
H15	0.139 (3)	−0.113 (2)	0.4405 (12)	0.039 (7)*	
H17	0.522 (3)	0.1020 (18)	0.4243 (12)	0.038 (7)*	
H18	0.424 (3)	−0.080 (2)	0.4398 (13)	0.052 (8)*	
H3A	0.020 (3)	0.9449 (18)	0.1915 (13)	0.037 (8)*	
H4A	0.487 (3)	0.2905 (19)	0.4204 (12)	0.044 (7)*	
H19A	0.515 (3)	0.4632 (17)	0.3747 (11)	0.025 (7)*	
H24A	0.072 (2)	0.8109 (17)	0.3210 (12)	0.029 (6)*	
H19B	0.392 (3)	0.5105 (18)	0.4335 (13)	0.035 (7)*	
H24B	0.025 (3)	0.7722 (17)	0.2349 (12)	0.029 (7)*	
N1	0.3482 (2)	0.55995 (16)	0.32678 (11)	0.0268 (5)	
N2	0.2136 (2)	0.69471 (16)	0.27795 (11)	0.0253 (5)	
N3	0.1147 (3)	0.96669 (17)	0.20510 (11)	0.0286 (5)	
N4	0.3760 (2)	0.28106 (17)	0.40204 (11)	0.0297 (5)	
O1	0.34999 (19)	0.89706 (13)	0.25021 (9)	0.0334 (4)	
O2	0.1733 (2)	0.37764 (14)	0.34706 (10)	0.0438 (5)	

### Atomic displacement parameters (Å^2^)

**Table:**

	*U*^11^	*U*^22^	*U*^33^	*U*^12^	*U*^13^	*U*^23^
C1	0.0260 (15)	0.0289 (16)	0.0266 (15)	−0.0041 (12)	0.0008 (12)	0.0001 (13)
C2	0.0312 (16)	0.0303 (16)	0.0317 (16)	0.0086 (14)	0.0023 (13)	−0.0032 (14)
C3	0.0300 (16)	0.0372 (17)	0.0288 (15)	0.0026 (13)	0.0001 (13)	0.0056 (14)
C4	0.0237 (15)	0.0287 (16)	0.0384 (17)	0.0032 (13)	0.0004 (14)	0.0028 (14)
C5	0.0216 (13)	0.0228 (14)	0.0289 (14)	0.0005 (11)	0.0042 (12)	−0.0003 (12)
C6	0.0289 (15)	0.0280 (16)	0.0347 (17)	0.0020 (13)	0.0004 (14)	−0.0004 (13)
C7	0.0296 (16)	0.0288 (17)	0.0523 (19)	0.0011 (13)	0.0042 (15)	−0.0083 (15)
C8	0.0396 (18)	0.0392 (19)	0.056 (2)	−0.0003 (16)	0.0123 (16)	0.0167 (17)
C9	0.0372 (17)	0.050 (2)	0.0388 (17)	−0.0016 (16)	−0.0035 (15)	0.0133 (16)
C10	0.0300 (16)	0.0366 (17)	0.0326 (16)	−0.0007 (14)	−0.0001 (13)	−0.0004 (14)
C11	0.0252 (15)	0.0331 (16)	0.0302 (14)	0.0024 (13)	0.0006 (12)	0.0030 (12)
C12	0.0329 (16)	0.0292 (16)	0.0227 (13)	0.0001 (13)	0.0021 (12)	0.0023 (12)
C13	0.0336 (17)	0.0351 (18)	0.0279 (14)	−0.0003 (14)	−0.0019 (13)	0.0040 (13)
C14	0.0385 (19)	0.047 (2)	0.0322 (16)	−0.0070 (16)	−0.0050 (14)	0.0011 (14)
C15	0.054 (2)	0.0349 (19)	0.0322 (16)	−0.0101 (17)	−0.0051 (15)	0.0017 (14)
C16	0.051 (2)	0.0366 (18)	0.0284 (15)	0.0083 (16)	−0.0033 (14)	0.0005 (13)
C17	0.0341 (17)	0.0357 (18)	0.0287 (15)	0.0039 (14)	0.0023 (13)	0.0000 (13)
C18	0.0247 (15)	0.0299 (16)	0.0276 (14)	0.0002 (13)	0.0011 (12)	−0.0029 (12)
C19	0.0251 (17)	0.0349 (17)	0.0341 (17)	0.0027 (13)	−0.0054 (14)	0.0062 (14)
Cl1	0.0346 (4)	0.0441 (4)	0.0375 (4)	0.0001 (3)	−0.0066 (3)	−0.0045 (3)
N1	0.0215 (11)	0.0291 (13)	0.0298 (12)	0.0011 (10)	−0.0009 (9)	0.0033 (10)
N2	0.0231 (11)	0.0233 (11)	0.0297 (11)	0.0004 (10)	0.0004 (10)	0.0031 (10)
N3	0.0215 (13)	0.0295 (13)	0.0348 (12)	−0.0016 (11)	−0.0041 (11)	0.0045 (10)
N4	0.0230 (12)	0.0335 (14)	0.0325 (12)	0.0013 (11)	−0.0047 (10)	0.0044 (11)
O1	0.0239 (10)	0.0311 (10)	0.0451 (11)	−0.0004 (8)	−0.0039 (9)	0.0031 (8)
O2	0.0294 (11)	0.0372 (12)	0.0644 (13)	−0.0015 (9)	−0.0159 (10)	0.0118 (9)

### Geometric parameters (Å, º)

**Table:**

C1—N1	1.329 (3)	C10—H10	0.99 (2)
C1—N2	1.329 (3)	C11—O2	1.225 (3)
C1—H1	0.97 (2)	C11—N4	1.340 (3)
C2—C3	1.340 (3)	C11—C19	1.523 (3)
C2—N1	1.382 (3)	C12—C17	1.384 (3)
C2—H2	0.91 (2)	C12—C13	1.391 (3)
C3—N2	1.371 (3)	C12—N4	1.426 (3)
C3—H3	0.91 (2)	C13—C14	1.387 (4)
C4—N2	1.451 (3)	C13—H13	0.97 (2)
C4—C18	1.522 (3)	C14—C15	1.375 (4)
C4—H24A	1.00 (2)	C14—H14	0.93 (2)
C4—H24B	0.98 (2)	C15—C16	1.374 (4)
C5—C6	1.378 (3)	C15—H15	1.00 (2)
C5—C10	1.392 (3)	C16—C17	1.392 (4)
C5—N3	1.415 (3)	C16—H18	0.99 (3)
C6—C7	1.387 (3)	C17—H17	1.00 (2)
C6—H6	0.94 (2)	C18—O1	1.223 (3)
C7—C8	1.382 (4)	C18—N3	1.343 (3)
C7—H7	0.94 (2)	C19—N1	1.464 (3)
C8—C9	1.376 (4)	C19—H19A	0.94 (2)
C8—H8	0.91 (2)	C19—H19B	0.99 (2)
C9—C10	1.368 (4)	N3—H3A	0.87 (2)
C9—H9	0.91 (2)	N4—H4A	0.99 (2)
			
N1—C1—N2	108.1 (2)	C13—C12—N4	123.6 (2)
N1—C1—H1	126.8 (12)	C14—C13—C12	119.2 (3)
N2—C1—H1	125.1 (12)	C14—C13—H13	118.9 (13)
C3—C2—N1	107.0 (2)	C12—C13—H13	121.8 (13)
C3—C2—H2	134.2 (13)	C15—C14—C13	121.2 (3)
N1—C2—H2	118.8 (13)	C15—C14—H14	124.2 (15)
C2—C3—N2	107.3 (2)	C13—C14—H14	114.6 (15)
C2—C3—H3	133.2 (14)	C16—C15—C14	119.4 (3)
N2—C3—H3	119.0 (14)	C16—C15—H15	121.0 (13)
N2—C4—C18	111.1 (2)	C14—C15—H15	119.6 (14)
N2—C4—H24A	107.4 (12)	C15—C16—C17	120.5 (3)
C18—C4—H24A	108.4 (13)	C15—C16—H18	123.5 (15)
N2—C4—H24B	107.8 (13)	C17—C16—H18	116.0 (15)
C18—C4—H24B	111.7 (12)	C12—C17—C16	119.9 (3)
H24A—C4—H24B	110.3 (18)	C12—C17—H17	117.5 (13)
C6—C5—C10	119.6 (2)	C16—C17—H17	122.6 (13)
C6—C5—N3	122.1 (2)	O1—C18—N3	126.0 (2)
C10—C5—N3	118.2 (2)	O1—C18—C4	121.1 (2)
C5—C6—C7	120.0 (2)	N3—C18—C4	112.9 (2)
C5—C6—H6	121.5 (14)	N1—C19—C11	108.6 (2)
C7—C6—H6	118.6 (14)	N1—C19—H19A	110.7 (13)
C8—C7—C6	120.1 (3)	C11—C19—H19A	111.0 (13)
C8—C7—H7	123.8 (14)	N1—C19—H19B	107.5 (13)
C6—C7—H7	116.0 (14)	C11—C19—H19B	109.0 (13)
C9—C8—C7	119.5 (3)	H19A—C19—H19B	109.9 (18)
C9—C8—H8	122.1 (15)	C1—N1—C2	108.6 (2)
C7—C8—H8	118.3 (15)	C1—N1—C19	125.1 (2)
C10—C9—C8	120.9 (3)	C2—N1—C19	125.9 (2)
C10—C9—H9	115.8 (16)	C1—N2—C3	108.9 (2)
C8—C9—H9	123.3 (16)	C1—N2—C4	126.6 (2)
C9—C10—C5	119.9 (3)	C3—N2—C4	124.5 (2)
C9—C10—H10	121.3 (12)	C18—N3—C5	126.0 (2)
C5—C10—H10	118.8 (12)	C18—N3—H3A	116.1 (15)
O2—C11—N4	124.6 (2)	C5—N3—H3A	115.7 (15)
O2—C11—C19	120.3 (2)	C11—N4—C12	126.5 (2)
N4—C11—C19	115.1 (2)	C11—N4—H4A	115.0 (14)
C17—C12—C13	119.7 (2)	C12—N4—H4A	118.5 (13)
C17—C12—N4	116.6 (2)		
			
N1—C2—C3—N2	−0.6 (3)	N2—C1—N1—C2	0.4 (3)
C10—C5—C6—C7	−1.0 (4)	N2—C1—N1—C19	−172.4 (2)
N3—C5—C6—C7	−178.1 (2)	C3—C2—N1—C1	0.1 (3)
C5—C6—C7—C8	1.8 (4)	C3—C2—N1—C19	172.8 (2)
C6—C7—C8—C9	−0.9 (4)	C11—C19—N1—C1	104.1 (3)
C7—C8—C9—C10	−0.8 (4)	C11—C19—N1—C2	−67.5 (3)
C8—C9—C10—C5	1.6 (4)	N1—C1—N2—C3	−0.8 (3)
C6—C5—C10—C9	−0.7 (4)	N1—C1—N2—C4	178.8 (2)
N3—C5—C10—C9	176.5 (2)	C2—C3—N2—C1	0.8 (3)
C17—C12—C13—C14	1.9 (4)	C2—C3—N2—C4	−178.7 (2)
N4—C12—C13—C14	−175.7 (2)	C18—C4—N2—C1	110.3 (3)
C12—C13—C14—C15	0.1 (4)	C18—C4—N2—C3	−70.2 (3)
C13—C14—C15—C16	−2.3 (4)	O1—C18—N3—C5	2.7 (4)
C14—C15—C16—C17	2.6 (4)	C4—C18—N3—C5	−179.6 (2)
C13—C12—C17—C16	−1.7 (4)	C6—C5—N3—C18	−45.1 (3)
N4—C12—C17—C16	176.1 (2)	C10—C5—N3—C18	137.8 (2)
C15—C16—C17—C12	−0.6 (4)	O2—C11—N4—C12	−4.3 (4)
N2—C4—C18—O1	−25.1 (3)	C19—C11—N4—C12	174.8 (2)
N2—C4—C18—N3	157.1 (2)	C17—C12—N4—C11	165.6 (2)
O2—C11—C19—N1	−22.6 (3)	C13—C12—N4—C11	−16.7 (4)
N4—C11—C19—N1	158.3 (2)		

### Hydrogen-bond geometry (Å, º)

**Table:**

*D*—H···*A*	*D*—H	H···*A*	*D*···*A*	*D*—H···*A*
N3—H3*A*···O2^i^	0.87 (2)	1.94 (2)	2.799 (3)	171 (2)
N4—H4*A*···Cl1^ii^	0.99 (2)	2.19 (2)	3.182 (2)	177.4 (19)
C1—H1···Cl1^iii^	0.97 (2)	2.58 (2)	3.482 (3)	155.0 (16)
C2—H2···O1^ii^	0.91 (2)	2.32 (2)	3.082 (3)	140.6 (17)
C13—H13···O2	0.97 (2)	2.29 (2)	2.862 (3)	116.9 (16)
C4—H24*B*···O2^i^	0.98 (2)	2.54 (2)	3.308 (3)	136.0 (16)

Symmetry codes: (i) −*x*, *y*+1/2, −*z*+1/2; (ii) −*x*+1, *y*−1/2, −*z*+1/2; (iii) *x*, −*y*+3/2, *z*+1/2.
